# Incidence and Clinical Characteristics of Drug-Induced Lung Disease Among Chemotherapy Recipients

**DOI:** 10.7759/cureus.63408

**Published:** 2024-06-28

**Authors:** Ruhsel Cörüt, Ozden Altundağ, Füsun Eyüboğlu

**Affiliations:** 1 Pulmonology, Giresun University Faculty of Medicine, Giresun, TUR; 2 Medical Oncology, Faculty of Medicine, Başkent University, Ankara, TUR; 3 Pulmonology, Faculty of Medicine, Başkent University, Ankara, TUR

**Keywords:** pulmonary symptoms, oncology, pulmonary toxicity, drug-induced lung disease, chemotherapy

## Abstract

Background

Chemotherapeutic agents treat cancer and some inflammatory diseases due to their immunosuppressive effects. While effective, these drugs can cause drug-induced lung disease (DILD), a serious adverse effect with limited data regarding its incidence and clinical presentation.

Methods

This retrospective study included 20 patients diagnosed with DILD out of 1,231 patients treated with chemotherapeutic agents who presented with symptoms such as cough, fever, dyspnea, and chest pain at an oncology outpatient clinic. Patients underwent assessments including clinical examination, chest radiography, high-resolution computed tomography, and, in some cases, video-assisted thoracoscopic surgery. A statistical analysis was performed to determine the incidence and evaluate the clinical characteristics of DILD.

Results

The incidence of DILD among patients treated with chemotherapeutic agents was 0.27%. The female/male ratio was 11/9, with a mean age of 53.2 years. Common symptoms included cough (70%), dyspnea (60%), fever (50%), and sputum production (40%). Imaging revealed pleural effusion, reticular patterns, and consolidation in varying proportions. Common agents causing pulmonary toxicity included bleomycin, cyclophosphamide, and methotrexate, among others. Importantly, 95% of patients showed improvement with steroid treatment, although statistical significance was not achieved (p > 0.05).

Conclusion

The findings highlight the need for heightened awareness and monitoring of DILD in patients receiving chemotherapeutic treatments. Early diagnosis and prompt treatment initiation are crucial to managing this potentially severe complication. This study underscores the importance of considering pulmonary risks when prescribing chemotherapeutic agents and provides foundational data for future research.

## Introduction

The therapeutic agents for combating cancer include chemotherapeutic drugs, known not only for their efficacy but also for their potential to cause significant organ toxicity. While the primary aim of chemotherapy is to maximize therapeutic effects, it is equally crucial to minimize associated toxicities [1]. Organ-specific toxicities, particularly pulmonary complications, represent a critical area of concern due to their potential severity and impact on patient morbidity and mortality [2].

Drug-induced lung diseases (DILDs) are increasingly recognized as a significant side effect of many pharmacological agents, including chemotherapeutics. These conditions range from benign infiltrates to severe, life-threatening lung diseases [3,4]. Despite numerous reports and growing recognition, the literature still lacks comprehensive data on the incidence and full spectrum of clinical presentations of DILD, compounded by challenges in diagnosis and underreporting [5]. The complexity of diagnosing DILD stems from its nonspecific symptoms and often requires advanced imaging techniques like high-resolution computed tomography (HRCT), which goes beyond routine chest radiography [6]. Furthermore, the vast array of chemotherapeutic agents contributes to a diverse variety of pulmonary pathologies, influenced by factors such as drug mechanism, patient-specific variables including age and previous exposure to chemotherapy, and genetic factors [7]. These agents can induce pulmonary toxicity acutely or over a more extended period, with symptoms ranging from mild cough and dyspnea to severe respiratory compromise [8].

Given the severe implications of DILD, early detection and prompt management are paramount. This involves discontinuing the offending agent and initiating treatments such as corticosteroids, which can significantly alter the disease course and improve outcomes [9,10]. However, the etiology, clinical course, and pathophysiology of chemotherapeutic-induced lung toxicity remain poorly understood, marked by direct and indirect effects on pulmonary tissue and immune responses.

This study aims to delineate the incidence, clinical characteristics, and outcomes of pulmonary toxicity among patients receiving chemotherapeutics at our center. By analyzing demographic data, drug dosage, treatment duration, and clinical outcomes, we seek to provide a detailed examination of pulmonary complications. We compare our findings with existing literature to underscore the significant impact of these toxicities and enhance understanding for better management and prevention strategies.

## Materials and methods

This retrospective study was approved by the Ethics Committee of the Başkent University Faculty of Medicine Research Board (approval number KA12/162). We analyzed DILD incidence and characteristics in patients presenting with respiratory symptoms at the Oncology Department Outpatient Clinic at Başkent University, Ankara, Turkey between 2008 and 2013.

We screened 1,231 patients presenting with cough, fever, shortness of breath, and chest pain. Patients were included if they were over 18 years of age and diagnosed with DILD based on clinical or radiological criteria. We excluded patients with positive cultures from sputum, bronchial lavage, or bronchoalveolar lavage (BAL); those with radiographic and clinical signs of infectious pneumonia, malignancy in the lung, or other specific lung pathologies like occupational lung disease, chemical pneumonitis, eosinophilic lung disease, and lung involvement secondary to cholangiogenic tissue disease. Two types of treatment were applied to patients with DILD. The first type of treatment was drug discontinuation, and the second was steroid administration.

Demographic data, chemotherapeutic dosages, durations, primary diagnoses, comorbidities, respiratory symptoms, and examination findings were recorded. This included arterial oxygen saturation and arterial blood gas analyses. We assessed the effects of chemotherapy on the primary disease course and explored associated toxicity, treatments, and outcomes.

Venous blood samples were analyzed using an Abbott Cell-Dyne® 3700 System in the Central Laboratory of Başkent University Medical Faculty Hospital. Standard parameters included leukocyte count, platelet count, sedimentation rate, and CRP levels. Pulmonary function tests (PFTs) were performed using a Sensormedics Vmax 221 spirometer and a portable Spirobank MIR spirometer. Measurements included forced vital capacity (FVC), forced expiratory volume in 1st second (FEV1), FEV1/FVC ratio, and diffusion capacity for carbon monoxide (DLCO).

Diagnostic imaging involved thorax CT, HRCT, and transthoracic biopsies as needed. Fiberoptic bronchoscopy was performed, followed by cytological examinations of BAL fluid in a subset of patients. Cells in BAL fluid were counted using a hemocytometer, and cellular distribution was assessed on smear preparations stained with May-Grünwald Giemsa (MGG). Normal and pathological findings were compared to established reference values for conditions such as alveolitis and pneumonitis, as indicated in Table 1 and Table 2.

**Table 1:** Normal BAL cell count rates BAL, bronchoalveolar lavage

Total count of cells	13 × 10^6^ mm^3^
Alveolar macrophage	84%
Lymphocyte	13%
Granulocyte	3%
Neutrophil	0.50%
Eosinophil	0.50%
Mast cell	0.50%
Plasma cell	0.50%

**Table 2:** Evaluation according to cell density in pathological MGG staining ARDS: acute respiratory distress syndrome; DILD: diffuse interstitial lung disease; MGG: May-Grünwald Giemsa; PAP: pulmonary alveolar proteinosis

Lymphocytic alveolitis	Neutrophilic alveolitis	Eosinophilic alveolitis
Hypersensitive pneumonia	Idiopathic pulmonary fibrosis	Eosinophilic pneumonia
Sarcoidosis	ARDS	Eosinophilic granulomatosis with polyangiitis
Berylliosis	Collagen vascular diseases	Allergic bronchopulmonary
Tuberculosis	Asbestosis	Aspergillosis
PAP	Pneumoconiosis	DILD
DILD	Bronchopulmonary infections	Bronchial asthma
Lymphangitis carcinomatosa		
Collagen vascular diseases		
Crohn’s disease		
AIDS		

Statistical analysis

Descriptive statistics of continuous values were expressed as frequency percentages (%). Fisher’s exact test and chi-square analysis were used to evaluate categorical data. The significance level was shown as p < 0.05. The data were evaluated in SPSS Statistics for Windows, Version 17.0 (Released 2008; SPSS Inc., Chicago, USA).

## Results

The study retrospectively included 20 cases diagnosed as DILD among 1,231 patients who presented to the oncology outpatient clinic with cough, fever, dyspnea, and chest pain. The male/female ratio was 11/9 in these cases. A total of 55% of the patients were male, and 45% were female. There was no statistically significant difference in the male-female distribution (p > 0.05). The ages of the patients varied between 18 and 80 years. The distribution of demographic characteristics of the patients according to gender is shown in Table 3.

**Table 3:** Demographic characteristics

n		%	Age (mean)	Smoke (average) (pack-year)
Male	11	55	60	12.1
Female	9	45	58	5.5
Total	20	100	59	9.2

The mean age of female patients was 58, and the mean age of male patients was 60. Female patients were between 31 and 75 years old, and male patients were between 18 and 80. The mean age of the patients was 59. Male patients had a maximum smoking history of 40 pack-years, and female patients had a maximum smoking history of 25 pack-years. The mean smoking history of all patients was 9.2 pack years.

Five patients had lung cancer, and 15 patients had other cancers. Among other cancers, five (25%) patients had breast cancer, six (30%) patients had leukemia, one (5%) patient had malignant melanoma, two (10%) patients had colon cancer, and one (5%) patient had a testicular tumor. The cancer type distribution of the patients is given in Table 4.

**Table 4:** Cancer-type rates of patients with DILD DILD: diffuse interstitial lung disease

Cancer type	Number of patients (n)	Percentage (%)
Lung cancer	5	25
Breast cancer	5	25
Leukemia	6	30
Malignant melanoma	2	10
Colon cancer	1	5
Testicular cancer	1	5

Ten patients presented to the oncology outpatient clinic with fever (50%), 14 with cough (70%), 12 with dyspnea (60%), eight with sputum (40%), and eight with other respiratory complaints (40%).

There was no statistically significant difference between respiratory complaints (p > 0.05). Table 5 shows the distribution of patient symptoms.

**Table 5:** Distribution of symptoms in our patients with DILD DILD: diffuse interstitial lung disease

Symptoms	Number of patients (n)	Percentage (%)	p
Cough	14	70	>0.05
Dyspnea	12	60	>0.05
Fever	10	50	>0.05
Sputum	8	40	>0.05
Other	8	40	>0.05

In our female patients, the highest arterial oxygen saturation was 98%, and the lowest was 82%. In our male patients, the highest arterial oxygen saturation was 97%, and the lowest saturation value was 75%. The mean saturation value of our patients was 89%. The mean partial pressure of oxygen (PaO2) was 59 mm Hg. The highest PaO2 was 77 mm Hg, and the lowest PaO2 was 45 in female patients. In male patients, the highest PaO2 was 80 mm Hg, and the lowest PaO2 was 42 mm Hg. No statistically significant difference was found (p > 0.05).

**Figure 1:**
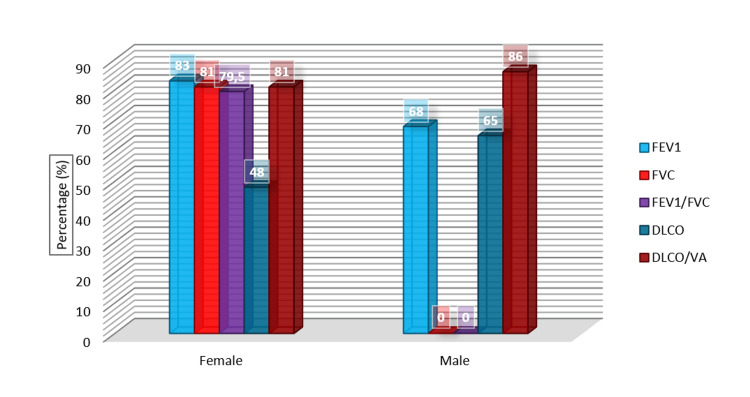
PFT and plethysmography results according to gender PFT: pulmonary function test

When PFTs performed on our patients were analyzed, the mean FEV1 percentage was 68%, the FVC percentage was 72.5%, the FEV1/FVC ratio was 78.5%, the DLCO was 65%, and the DLCO/VA was 86% in our male patients. In our female patients, the mean FEV1 percentage was 83%, the FVC percentage was 81%, the FEV1/FVC ratio was 79.5%, the DLCO was 48%, and the DLCO/VA was 81%. In all our patients, the mean FEV1 percentage was 69%, the FVC percentage was 73%, the FEV1/FVC ratio was 79%, DLCO was 63%, and DLCO/VA was 86%. No statistically significant difference was found between these data (p > 0.05). Figure 1 shows the PFT and plethysmography results according to gender for our patients receiving chemotherapy.

When the preliminary diagnoses of these 20 patients were analyzed, DILD was considered in nine patients, pneumonia in eight patients, and pleural effusion in three patients. DILD was treated with a prediagnosis of DILD in 45%, pneumonia in 40%, and pleural effusion in 15% of the patients.

Direct chest radiography revealed pleural effusion in five patients, reticular appearance in 10 patients, and consolidation in five. While reticular appearances were numerically more common, there was no statistically significant difference between all appearances on chest radiography (p > 0.05). Table 6 shows the distribution of the appearance of patients with DILD chest radiographs.

**Table 6:** Distribution of radiological findings in patients with DILD DILD: diffuse interstitial lung disease

X-ray image findings	Number of patients (n)	Percentage (%)	p
Reticular findings	10	50	>0.05
Pleural effusion	5	25	>0.05
Consolidation	5	25	>0.05
HRCT findings	Number of patients (n)	Percentage (%)	p
Normal	5	25	>0.05
Ground glass pattern	6	30	>0.05
Ground glass + fibrosis	9	45	>0.05

HRCT was performed in all cases. Six patients (25%) had ground glass, nine patients (45%) had ground glass + interstitial pattern + fibrosis, and five patients (30%) had no pathology on CT. The distribution of this appearance was not statistically significant (p > 0.05). Table 6 shows the HRCT appearance distribution of patients with DILD.

Twenty patients underwent fiberoptic bronchoscopy. The results were compatible with DILD in 13 patients (65%) and not with DILD in seven (35%). Among the seven cases compatible with DILD, two patients (10%) had lymphocytic alveolitis, one patient (5%) had eosinophilic alveolitis, and four patients (20%) had fibrosis on cytology. This concordance was not statistically significant. The agreement rate distribution of fiberoptic bronchoscopy with DILD is shown in Table 7.

**Table 7:** Agreement rate distribution of fiberoptic bronchoscopy with DILD DILD: diffuse interstitial lung disease; MGG: May-Grünwald Giemsa

Bronchoscopic examination	Number of patients (n)	Percentage (%)	p
Yes	7	35	>0.05
No	13	65	>0.05
Cell counting in MGG staining			
Lymphocytic alveolitis	2	10	>0.05
Eosinophilic alveolitis	1	5	>0.05
Fibrosis in biopsy	4	20	>0.05

Two of the patients were diagnosed with DILD by VATS, while 18 were diagnosed by clinic and radiology.

A total of 40% of the patients were receiving radiotherapy concurrently with chemotherapy. Twelve patients did not receive radiotherapy. No statistically significant difference was found (p > 0.05). When the contribution of radiotherapy was analyzed in DILD patients receiving chemotherapy, no statistically significant difference was observed compared to patients who did not receive radiotherapy.

**Figure 2:**
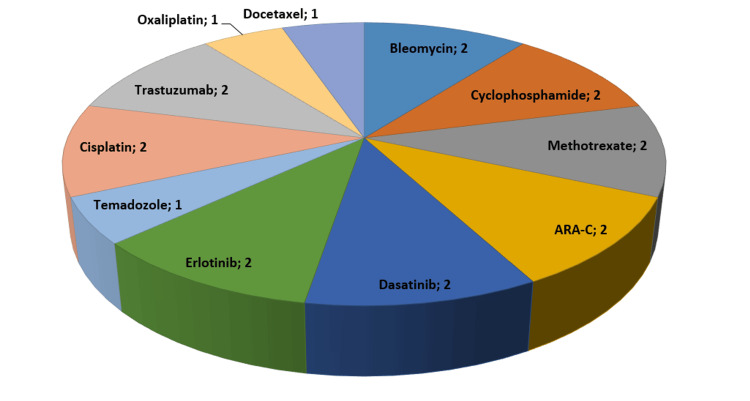
Distribution of chemotherapy drugs causing DILD diagnoses among cases DILD: diffuse interstitial lung disease

In our study, we diagnosed DILD due to chemotherapeutic agents that cause the most pulmonary toxicity, such as bleomycin (two patients), cyclophosphamide (two patients), methotrexate (two patients), ARA-C (two patients), dasatinib (two patients), erlotinib (two patients), temadozole (one patient), cisplatin (two patients), trastuzumab (two patients), oxaliplatin (one patient), and docetaxel (one patient). Figure 2 shows the distribution of chemotherapeutic agents causing DILD diagnosis.

Two types of treatment were applied to DILD patients. The first type of treatment was drug withdrawal, and the second was steroid administration. Only one of our patients benefited from drug withdrawal. The other 19 patients benefited from steroid treatment with methylprednisolone at a dose of 0.5-1 mg/kg or bioequivalent corticosteroids. The results showed that steroid treatment for our patients was statistically significant compared to chemotherapeutic agent discontinuation treatment (p < 0.05).

## Discussion

The annual incidence of DILD identified in our study is 0.27%, underscoring the importance of vigilance when monitoring patients receiving chemotherapeutic agents. This incidence aligns with findings from other studies, such as those conducted by Liu et al., who reported a similar incidence rate [11]. This indicates that DILD remains a significant clinical concern across diverse patient populations and treatment protocols, despite its relative rarity.

The clinical presentation of our patients, primarily cough, dyspnea, and fever, is consistent with the symptoms highlighted in the literature as common indicators of DILD, as noted by Skeoch et al. [12]. Our reliance on HRCT for diagnosing these cases reflects current best practices and is supported by Tamura et al., who affirm HRCT’s diagnostic superiority over conventional chest radiography [13]. This diagnostic approach is crucial for accurately detecting and appropriately managing chemotherapy-associated pulmonary complications.

One of the significant findings from our research is the efficacy of corticosteroids in managing DILD, with 95% of our patients showing clinical improvement following treatment. Melani et al. documented similar benefits of steroids, reinforcing the potential of early therapeutic intervention to substantially modify the disease trajectory [14]. Such results highlight the necessity of prompt recognition and treatment of pulmonary toxicity, which can dramatically improve patient outcomes.

Moreover, our study paves the way for future research directions, such as the development of biomarkers for early detection of DILD. Recent advancements in genomic and proteomic research suggest that identifying specific biomarkers could significantly improve the management of patients treated with chemotherapeutic agents. Additionally, understanding genetic predispositions to DILD, as explored by Steele and Brown [15], could lead to more personalized and effective treatments, reducing the risk of severe pulmonary outcomes [16,17].

Despite its contributions, our study is not without limitations, primarily due to its retrospective nature and the relatively small sample size. These factors may limit the generalizability of our results. However, the detailed clinical and diagnostic evaluations strengthen our findings’ validity and provide a meaningful addition to the limited body of literature on this topic. Our research corroborates earlier studies on the pulmonary risks associated with chemotherapeutic agents and highlights the crucial role of early diagnosis and intervention. Embracing more personalized approaches to chemotherapy, with an acute awareness of potential pulmonary toxicity, represents a promising advancement in the field of oncology.

## Conclusions

This study has delineated the incidence and characteristics of DILD among chemotherapy patients, underscoring the necessity for careful monitoring and early intervention. Our findings affirm the value of HRCT in diagnosing DILD and highlight the influential role of corticosteroids in treatment, significantly enhancing patient outcomes. As chemotherapy continues to be a mainstay in cancer treatment, our research stresses the importance of a nuanced understanding of its potential pulmonary risks. Future investigations should focus on biomarkers for early detection and genetic factors predisposing individuals to DILD, promising advancements that could lead to more personalized and safer therapeutic strategies. Through such efforts, we aim to refine our approach to chemotherapy, optimizing efficacy while minimizing adverse effects.
